# Nomogram to diagnosis of obstructive sleep apnoea‐hypopnoea syndrome in high‐risk Chinese adult patients

**DOI:** 10.1111/crj.13682

**Published:** 2023-08-02

**Authors:** Jie Liu, Feng Pang, Xiaofeng Huang, Xiangmin Zhang, Minmin Lin, Wenmin Deng, Tianrun Liu, Zhen Long

**Affiliations:** ^1^ Department of Otorhinolaryngology Head and Neck Surgery, Department of Sleep Medicine The Sixth Affiliated Hospital, Sun Yat‐sen University Guangzhou China

**Keywords:** cross‐sectional studies, nomograms, obstructive, sleep apnoea, sleep apnoea syndromes

## Abstract

**Introduction:**

Many scales are designed to screen for obstructive sleep apnoea‐hypopnoea syndrome (OSAHS); however, there is a lack of an efficiently and easily diagnostic tool, especially for Chinese. Therefore, we conduct a cross‐sectional study in China to develop and validate an efficient and simple clinical diagnostic model to help screen patients at risk of OSAHS.

**Methods:**

This study based on 782 high‐risk patients (aged >18 years) admitted to the Sleep Medicine department of the Sixth Affiliated Hospital, Sun Yat‐sen University from 2015 to 2021. Totally 34 potential predictors were evaluated. We divided all patients into training and validation dataset to develop diagnostic model. The univariable and multivariable logistic regression model were used to build model and nomogram was finally built.

**Results:**

Among 602 high‐risk patients with median age of 46 (37, 56) years, 23.26% were women. After selecting using the univariate logistic model, 15 factors were identified. We further used the stepwise method to build the final model with five factors: age, BMI, total bilirubin levels, high Berlin score, and symptom of morning dry mouth or mouth breathing. The AUC was 0.780 (0.711, 0.848), with sensitivity of 0.848 (0.811, 0.885), specificity of 0.629 (0.509, 0.749), accuracy of 0.816 (0.779, 0.853). The discrimination ability had been verified in the validation dataset. Finally, we established a nomogram model base on the above final model.

**Conclusion:**

We developed and validated a predictive model with five easily acquire factors to diagnose OSAHS patient in high‐risk population with well discriminant ability. Accordingly, we finally build the nomogram model.

## Graphical abstract

This cross‐sectional study was to develop and validate an efficient and simple clinical diagnostic model to help screen patients at risk of OSAHS. Totally 34 potential predictors were evaluated. The final model has been built with five factors: age, BMI, total bilirubin levels, high Berlin score, and symptom of morning dry mouth or mouth breathing.

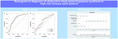

## 1 INTRODUCTION

Obstructive sleep apnoea‐hypopnoea syndrome (OSAHS) is the most common sleep‐related breathing disorder, which is characterized by periodic, partial, or complete collapse of the upper airway during sleep, resulting in brief sleep arousals to restore airway patency.^1^ In the general population, the prevalence of OSAHS ranges from 9% to 38% and increases as age and body mass index, especially in males.^2^ A recent study found that obesity and OSAS adversely affect many organs and systems. Besides the factors affecting the diagnosis of the OSAS‐obesity relationship, mutual organ interactions among the respiratory system, adipose tissue and intestines should not be ignored for prevention and treatment of OSAS and obesity.^3^ Untreated OSAHS is usually associated with adverse long‐term health outcomes, such as systemic hypertension, pulmonary hypertension, heart failure, cardiac arrhythmias, diabetes mellitus, and stroke.^4^, ^5^, ^6^ OSAHS is also associated with poorer health‐related quality of life and higher cost of healthcare resources.^7^, ^8^, ^9^ Recently, a special issue of Frontiers in Medicine for ‘Obstructive sleep apnoea syndrome (OSAS). What's new?’ also collected many recent researches about the OSAS, which is linked with a high risk of hypertension, cardiovascular diseases, daytime sleepiness, home and work‐related accidents, and a consequent worsening of life quality. Numerous studies stress the importance of praecox diagnosis and a multidisciplinary approach in addressing all these situations.^10^

Currently, public awareness of OSAHS is increasing, and more and more high‐risk patients are willing to evaluate the disease and seek treatment opportunities, but OSAHS remains severely underdiagnosed,^11^ so that a large proportion of patients do not receive any appropriate treatment, even though have no idea of this disease. At present, the gold standard for diagnosing OSAHS is polysomnography (PSG).^12^ However, the measurement of PSG requires the patient to stay overnight in the sleep monitoring centre. The PSG is time‐consuming and expensive, additionally not all medical institutions have sleep monitoring centers and well‐trained and professional sleep physicians. With the rising incidence of OSAHS due to rising obesity rates and an aging population, there is an urgent need to develop a simple and effective diagnostic tool that could allow for a wider screening and early diagnosis of OSAHS.

A lot of scales have been designed and developed to screen for OSAHS patients, including Espworth sleepiness scale (ESS), STOP‐Bang and Berlin score. For example, a recent study found that a non‐invasive assessment of low ArTH could help to identify an endotype with a lower predicted response to oral appliances in a clinical setting.^13^ However, previous studies shown that the specificity and negative predictive value (NPV) of these scales were not high, less than 60%,^14^ especially in the Chinese population.^15^ These scales cannot effectively discriminate the non‐OSAHS patients and lead to a large proportion of unnecessary PSG screening. Therefore, we conduct a cross‐sectional study in a Chinese population with high‐risk to develop and validate a practical clinical model to screen patients of OSAHS.

## 2 MATERIALS AND METHODS

### 2.1 Patients

This is a cross‐sectional study including all patients with suspected OSAHS admitted in respiratory care department of The Sixth Affiliated Hospital, Sun Yat‐sen University from 2015 to 2021. The patients who were aged 18 years or older and suspected OSAHS patients admitted for polysomnography examination due to snoring or other related symptoms. The patients who were pregnant, had received OSAHS treatment in the past, or had mental illness and currently used the sedatives or antipsychotics, were excluded from this study. This study was approved by Ethics Committee of The Sixth Affiliated Hospital Sun Yat‐sen University (2023ZSLYEC‐001).

### 2.2 Diagnosis of OSAHS

The OSAHS was defined if apnoea‐hypopnoea index (AHI) assessed by polysomnography was ≥5/h. After admission, all patients received polysomnography for an overnight monitoring. We invited two professional physicians with more than 5 years of experience to blindly and independently evaluate the monitoring data of polysomnography for each patient.

### 2.3 Selection and measurements of predictive factors

The potential predictors of OSAHS were included according to a systematic review of the current published literature in resent 10 years. We also invited several experts in this area to help us to evaluate and select the possible predictors for OSAHS. All these characteristics were collected at admission, which were abstracted and used as candidate predictors, including age, gender, OSAHS‐related symptoms, ESS, blood pressure, BMI, lifestyle information, comorbidities, and circulating biochemical markers.

Finally, we selected the potential risk factors including the general factors (age, gender, and body mass index [BMI]), lifestyle factors (current smoking status and drinking status), the clinical syndrome of OSAHS (obstructive sleep apnoea, morning dry mouth and mouth breathing, heart palpitations at night, nocturia, and waking up dizzy or morning headaches), systolic and diastolic blood pressure, comorbidities (diabetes, hypertension, thyroid hypofunction, cardiovascular heart disease, and gastroesophageal reflux disease), ESS, AHI, and Berlin score as the potential predictors in this study. All above information was collected and recorded by study technicians or extracted from the electronic medical records after masking the confidential information of patients. Additionally, the circulating biochemical biomarkers, including fasting plasma glucose, total cholesterol, high‐density lipoprotein cholesterol, low‐density lipoprotein cholesterol, triglycerides, aspartate aminotransferase, alanine aminotransferase, gamma‐glutamyl transferase, alkaline phosphatase, prothrombin, albumin, platelet count, and total bilirubin, were assessed in the central laboratory of the Sixth Affiliated Hospital, Sun Yat‐sen University under the standard operation procedure. All data were double checked by the independent data reviewers to reconfirm the accuracy of the data; if any inconsistency we will discuss and finally confirm the data.

### 2.4 Statistical analyses

We divided all patients (*n* = 782) into two datasets according to the year of admission: the training dataset included the patients from 2015 to 2018 with 602 patients; while the validation dataset with 180 patients were from 2019 to 2021. The training dataset was used to establish the diagnosis model and the validation dataset was applied to validate the established model.

We calculated the mean (±standard deviation [SD]) or median (the 25th percentile and 75th percentile) for continuous variables and count (percentage %) for categorical variables, whenever appreciate. The student's t test for normally distributed data or Mann–Whitney *U* test for data with skewed distribution was used to compare the difference in characteristics between training and validation datasets or OSAHS or non‐OSAHS patients in training dataset. Kolmogorov–Smirnov test was applied to evaluate the normal distribution of continuous variables. Binary or categorical variables were tested by using chi‐square tests or Fisher's exact test.

Using the training dataset, we firstly evaluated and selected the individual associations between potential characteristics and OSAHS by using the univariate logistic regression model. The odds ratio (OR) and 95% confidential intervals (CIs) of different predictors were calculated. The statistically significant (*P* < 0.05) factors were selected and used for further selection by using multivariable logistic regression model. We used the stepwise selection method to help us select the predictor factors and finally built the diagnosis model for OSAHS. We built three best models for final selection. Based on the finally model, we established a nomogram model to rate the diagnosis value.

We used the receiver operator characteristic (ROC) curve and calculating the integrated area under the ROC curve (AUC) and its 95% CI to evaluate the discrimination of the diagnosis model. We also calculated the sensitivity, specificity, positive/negative predictive value, accuracy, positive/negative likelihood ratio (LR) and their 95% CIs to assess the discrimination of the diagnosis model. Use the DeLongs test, we compared the difference in AUC values between three different models and decided the final model.

Using the validation dataset, we verified the previously established diagnostic model, and evaluated the performance of the diagnostic models by checking the model discrimination and calibration. All above statistical values were estimated to reevaluate the performance of the diagnostic model.

All statistical analyses were applied by using the SAS, version 9.4 (SAS Institute, Inc. Cary, NC, USA). A two‐sided *P*‐value < 0.05 was considered as statistically significant.

## 3 RESULTS

A total of 782 patients aged 46 years (37–55 year) with high‐risk of OSAHS were met the inclusion criteria and enrolled into this study (Table 1). Among them, 23.02% (*n* = 180) were women. Most of the patients' characteristics between training and validation datasets were comparable, only several laboratory tests, such as high‐density lipoprotein, low‐density lipoprotein, prothrombin, albumin, and platelet count, Berlin score, and age were slightly different between two groups of patients (all *P*‐values > 0.05). Compared with non‐OSAHS patients, those finally diagnosed OSAHS patients were more frequently to be men (79.24% vs. 64.36%), with older age (47 vs. 39.5 years), current drinkers (36.13% vs. 23.23%), with high blood pressures, with the symptom of morning dry mouth/mouth breathing and nocturia, higher AHI, ESS, and Berlin score, higher levels of fasting glucose, triglycerides, alanine aminotransferase, gamma‐glutamyl transferase, and alkaline phosphatase; and lower levels of total bilirubin (all *P*‐values < 0.05) (Table 2).

**TABLE 1:** Characteristics of participants in training and validation dataset.

Characteristics	Total (*n* = 782)	Training dataset (*n* = 602)	Validation dataset (*n* = 180)	*P*‐value
Females	180 (23.02)	140 (23.26)	40 (22.22)	0.7726
Age, years	46 (37, 55)	46 (37, 56)	44 (35, 53)	0.0147
Current smokers	275 (35.26)	204 (34.00)	71 (39.44)	0.1799
Current drinkers	271 (34.70)	209 (34.78)	62 (34.44)	0.9348
BMI, kg/m^2^	26.72 ± 4.04	26.65 ± 4.1	26.94 ± 3.83	0.3896
SBP, mmHg	134.57 ± 14.19	134.36 ± 14.03	135.29 ± 14.74	0.4368
DBP, mmHg	87.69 ± 10.68	87.24 ± 10.28	89.17 ± 11.82	0.0498
History of diabetes	101 (12.93)	85 (14.14)	16 (8.89)	0.0654
History of hypertension	240 (30.73)	191 (31.78)	49 (27.22)	0.2449
History of CHD	63 (8.07)	54 (8.99)	9 (5.00)	0.085
History of thyroid hypofunction	43 (5.51)	29 (4.83)	14 (7.78)	0.1276
History of GERD	181 (23.18)	135 (22.46)	46 (25.56)	0.3883
Symptom				
Obstructive sleep apnoea	363 (46.60)	278 (46.41)	85 (47.22)	0.8482
Morning dry mouth/mouth breathing	708 (90.65)	541 (90.02)	167 (92.78)	0.2643
Heart palpitations at night	161 (20.61)	120 (19.97)	41 (22.78)	0.4135
Nocturia	311 (39.87)	247 (41.17)	64 (35.56)	0.1775
Waking up dizzy/morning headaches	270 (34.66)	214 (35.73)	56 (31.11)	0.2539
AHI (times/h)	24.6 (8.8, 56.9)	25 (8.8, 56.3)	23.35 (9.2, 58.95)	0.7954
ESS	8 (5, 12)	8 (5, 12)	8 (5, 11)	0.9633
High Berlin score	525 (71.43)	382 (68.83)	143 (79.44)	0.0062
Fasting plasma glucose, mmol/L	5.21 (4.82, 5.94)	5.23 (4.83, 5.96)	5.18 (4.81, 5.83)	0.2551
Total cholesterol, mg/dL	5.36 ± 1.18	5.37 ± 1.14	5.31 ± 1.31	0.5969
HDL‐cholesterol, mg/dL	1.28 ± 0.38	1.32 ± 0.4	1.15 ± 0.25	<0.0001
LDL‐cholesterol, mg/dL	3.21 ± 0.87	3.13 ± 0.88	3.46 ± 0.78	<0.0001
Triglycerides, mg/dL	1.76 (1.22, 2.64)	1.76 (1.22, 2.59)	1.71 (1.25, 2.76)	0.9444
AST, IU/L	21.2 (17.73, 27.54)	21.56 (17.77, 28)	20.59 (17.62, 26.86)	0.577
ALT, IU/L	27.52 (18.89, 42)	27.99 (18.6, 41.47)	26.79 (19.19, 45.23)	0.8641
GGT, IU/L	33 (24, 53.33)	33 (24, 53)	34.07 (23.54, 53.55)	0.9629
ALP, IU/L	79.25 ± 23.9	79.11 ± 23.57	79.74 ± 25.03	0.7718
PT, s	11.6 (11.12, 12.18)	11.72 (11.27, 12.24)	11.3 (10.8, 11.8)	<0.0001
Albumin, g/dL	43.51 (41.2, 46.02)	43.81 (41.5, 46.36)	42.42 (40.28, 45.25)	0.0002
Platelet count, 10^9^/L	231.99 ± 60.78	223.22 ± 57.18	261.54 ± 63.35	<0.0001
Total bilirubin, mg/dL	12.87 ± 5.27	12.75 ± 5.28	13.21 ± 5.23	0.342

Abbreviations: AHI, apnoea‐hypopnoea index; ALP, alkaline phosphatase; ALT, alanine aminotransferase; AST, aspartate aminotransferase; BMI, body mass index; CHD, cardiovascular heart disease; DBP, diastolic blood pressure; ESS, Espworth sleepiness scale; GERD, gastroesophageal reflux disease; GGT, gamma‐glutamyl transferase; HDL, high‐density lipoprotein; LDL, low‐density lipoprotein; PT, prothrombin; SBP, systolic blood pressure.

**TABLE 2:** Characteristics of OSAHS and non‐OSAHS participants in training dataset.

Characteristics	Total (*n* = 602)	OSAHS (*n* = 501)	Non‐OSAHS (*n* = 101)	*P*‐value
Females	140 (23.26)	104 (20.76)	36 (35.64)	0.0012
Age, years	46 (37, 56)	47 (39, 57)	39.5 (32, 51)	<0.0001
Current smokers	209 (34.78)	181 (36.06)	28 (28.28)	0.1378
Current drinkers	204 (34.00)	181 (36.13)	23 (23.23)	0.0133
BMI, kg/m^2^	26.65 ± 4.1	27.13 ± 4.01	24.2 ± 3.65	<0.0001
SBP, mmHg	134.36 ± 14.03	135.44 ± 13.7	128.84 ± 14.44	<0.0001
DBP, mmHg	87.24 ± 10.28	88.15 ± 10.15	82.67 ± 9.74	<0.0001
History of diabetes	85 (14.14)	76 (15.14)	9 (9.09)	0.1145
History of hypertension	191 (31.78)	174 (34.66)	17 (16.47)	0.0006
History of CHD	54 (8.99)	45 (8.96)	9 (9.09)	0.9678
History of thyroid hypofunction	29 (4.83)	24 (4.78)	5 (5.05)	0.9089
History of GERD	135 (22.46)	112 (22.31)	23 (23.23)	0.8409
Symptom				
Obstructive sleep apnoea	278 (46.41)	239 (47.70)	39 (39.80)	0.1511
Morning dry mouth/mouth breathing	541 (90.02)	461 (91.83)	80 (80.81)	0.0008
Heart palpitations at night	120 (19.97)	99 (19.72)	21 (21.21)	0.7345
Nocturia	247 (41.17)	216 (43.11)	31 (31.31)	0.0292
Waking up dizzy/morning headaches	214 (35.73)	180 (36.00)	34 (34.34)	0.7533
AHI (times/h)	25 (8.8, 56.3)	37.2 (15, 61)	1.4 (0.7, 2.8)	<0.0001
ESS	8 (5, 12)	8 (5, 12)	7 (4, 10)	0.0335
High Berlin score	382 (68.83)	344 (75.27)	38 (38.78)	<0.0001
Fasting plasma glucose, mmol/L	5.23 (4.83, 5.96)	5.28 (4.87, 6.02)	4.99 (4.75, 5.38)	0.0006
Total cholesterol, mg/dL	5.37 ± 1.14	5.4 ± 1.13	5.18 ± 1.17	0.1389
HDL‐cholesterol, mg/dL	1.32 ± 0.4	1.31 ± 0.4	1.4 ± 0.41	0.0798
LDL‐cholesterol, mg/dL	3.13 ± 0.88	3.15 ± 0.88	3.03 ± 0.87	0.284
Triglycerides, mg/dL	1.76 (1.22, 2.59)	1.79 (1.25, 2.64)	1.5 (1.01, 2.35)	0.0207
AST, IU/L	21.56 (17.77, 28)	22 (17.97, 28)	20.06 (17, 26.1)	0.0775
ALT, IU/L	27.99 (18.6, 41.47)	29 (19.64, 42.62)	22.16 (16.48, 32)	0.0013
GGT, IU/L	33 (24, 53)	34.2 (25.07, 55.52)	26 (19, 34.7)	<0.0001
ALP, IU/L	79.11 ± 23.57	79.98 ± 23.93	74.17 ± 20.88	0.046
PT, s	11.72 (11.27, 12.24)	11.72 (11.27, 12.24)	11.76 (11.31, 12.24)	0.5306
Albumin, g/dL	43.81 (41.5, 46.36)	43.67 (41.47, 46.2)	44.3 (41.65, 47.67)	0.1601
Platelet count, 10^9^/L	223.22 ± 57.18	222.62 ± 56.87	226.28 ± 59	0.5689
Total bilirubin, mg/dL	12.75 ± 5.28	12.41 ± 4.62	14.78 ± 7.94	0.0208

Abbreviations: AHI, apnoea‐hypopnoea index; ALP, alkaline phosphatase; ALT, alanine aminotransferase; AST, aspartate aminotransferase; BMI, body mass index; CHD, cardiovascular heart disease; DBP, diastolic blood pressure; ESS, Espworth sleepiness scale; GERD, gastroesophageal reflux disease; GGT, gamma‐glutamyl transferase; HDL, high‐density lipoprotein; LDL, low‐density lipoprotein; OSAHS, obstructive sleep apnoea‐hypopnoea syndrome; PT, prothrombin; SBP, systolic blood pressure.

The univariate logistic regression model selected 15 factors from totally 34 factors were significantly related with OSAHS with all *P*‐values < 0.05 (Table 3). Then, using the stepwise multivariate logistic regression model, we obtained the final model, including age (1.032 [95% CI: 1.010, 1.056], *P* = 0.005), BMI (1.221 [1.109, 1.345], *P* < 0.001), symptom of morning dry mouth/mouth breathing (3.033 [1.381, 6.663], *P* = 0.0057), Berlin score (2.154 [1.165, 3.983], *P* = 0.0144), and total bilirubin (0.939 [0.891, 0.989], *P* = 0.0179). Considering the previous evidence, we finally selected three models: Model 1: age, BMI, total bilirubin, symptom of morning dry mouth/mouth breathing, and Belin scores, Model 2: age, gender, BMI, total bilirubin, and symptom of morning dry mouth/mouth breathing, and Model 3: age, gender, BMI, total bilirubin, symptom of morning dry mouth/mouth breathing, and Berlin score.

**TABLE 3:** Univariate and multivariable model selection of risk factors for OSAHS in training dataset.

Characteristics	Univariate models	*P*‐value	Multivariable model	*P*‐value
	OR (95% CI)		OR (95% CI)	
Females	0.473 (0.298, 0.750)	0.0015		
Age, years	1.036 (1.018, 1.054)	<0.0001	1.032 (1.010, 1.056)	0.005
BMI, kg/m^2^	1.252 (1.169, 1.340)	<0.0001	1.221 (1.109, 1.345)	<0.0001
Current smokers	1.869 (1.133, 3.084)	0.0144		
Current drinkers	1.430 (0.890, 2.296)	0.1393		
SBP, mm Hg	1.037 (1.020, 1.055)	<0.0001		
DBP, mm Hg	1.061 (1.035, 1.87)	<0.0001		
History of diabetes	1.784 (0.862, 3.692)	0.1188		
History of hypertension	2.559 (1.471, 4.451)	0.0009		
History of CHD	0.930 (0.471, 1.835)	0.8335		
History of thyroid hypofunction	1.233 (0.509, 2.985)	0.6421		
History of GERD	0.949 (0.569, 1.583)	0.8409		
Symptom				
Obstructive sleep apnoea	1.380 (0.888, 2.144)	0.1522		
Morning dry mouth/mouth breathing	2.670 (1.475, 4.834)	0.0012	3.033 (1.381, 6.663)	0.0057
Heart palpitations at night	0.912 (0.537, 1.550)	0.7345		
Nocturia	1.662 (1.049, 2.634)	0.0304		
Waking up dizzy/morning headaches	1.075 (0.683, 1.692)	0.7534		
AHI (times/h)	67.03 (5.519, 814)	0.001		
ESS	1.047 (1.003, 1.093)	0.0346		
High Berlin Score	4.807 (3.039, 7.603)	<0.0001	2.154 (1.165, 3.983)	0.0144
Fasting plasma glucose, mmol/L	1.129 (0.992, 1.286)	0.0665		
Total cholesterol, mg/dL	1.195 (0.944, 1.514)	0.1394		
HDL‐cholesterol, mg/dL	0.611 (0.348, 1.072)	0.0859		
LDL‐cholesterol, mg/dL	1.113 (0.863, 1.436)	0.408		
Triglycerides, mg/dL	0.994 (0.840, 1.175)	0.9415		
AST, IU/L	1.006 (0.992, 1.022)	0.3956		
ALT, IU/L	1.009 (0.998, 1.020)	0.1027		
GGT, IU/L	1.019 (1.007, 1.031)	0.0014		
ALP, IU/L	1.012 (1.000, 1.023)	0.0455		
PT, s	1.010 (0.974, 1.047)	0.6043		
Albumin, g/dL	0.997 (0.991, 1.004)	0.4262		
Platelet count, 10^9^/L	0.999 (0.995, 1.003)	0.5683		
Total bilirubin, mg/dL	0.933 (0.893, 0.975)	0.0019	0.939 (0.891, 0.989)	0.0179

Abbreviations: AHI, apnoea‐hypopnoea index; ALP, alkaline phosphatase; ALT, alanine aminotransferase; AST, aspartate aminotransferase; BMI, body mass index; CHD, cardiovascular heart disease; DBP, diastolic blood pressure; ESS, Espworth sleepiness scale; GERD, gastroesophageal reflux disease; GGT, gamma‐glutamyl transferase; HDL, high‐density lipoprotein; LDL, low‐density lipoprotein; OSAHS, obstructive sleep apnoea‐hypopnoea syndrome; PT, prothrombin; SBP, systolic blood pressure.

**FIGURE 1:**
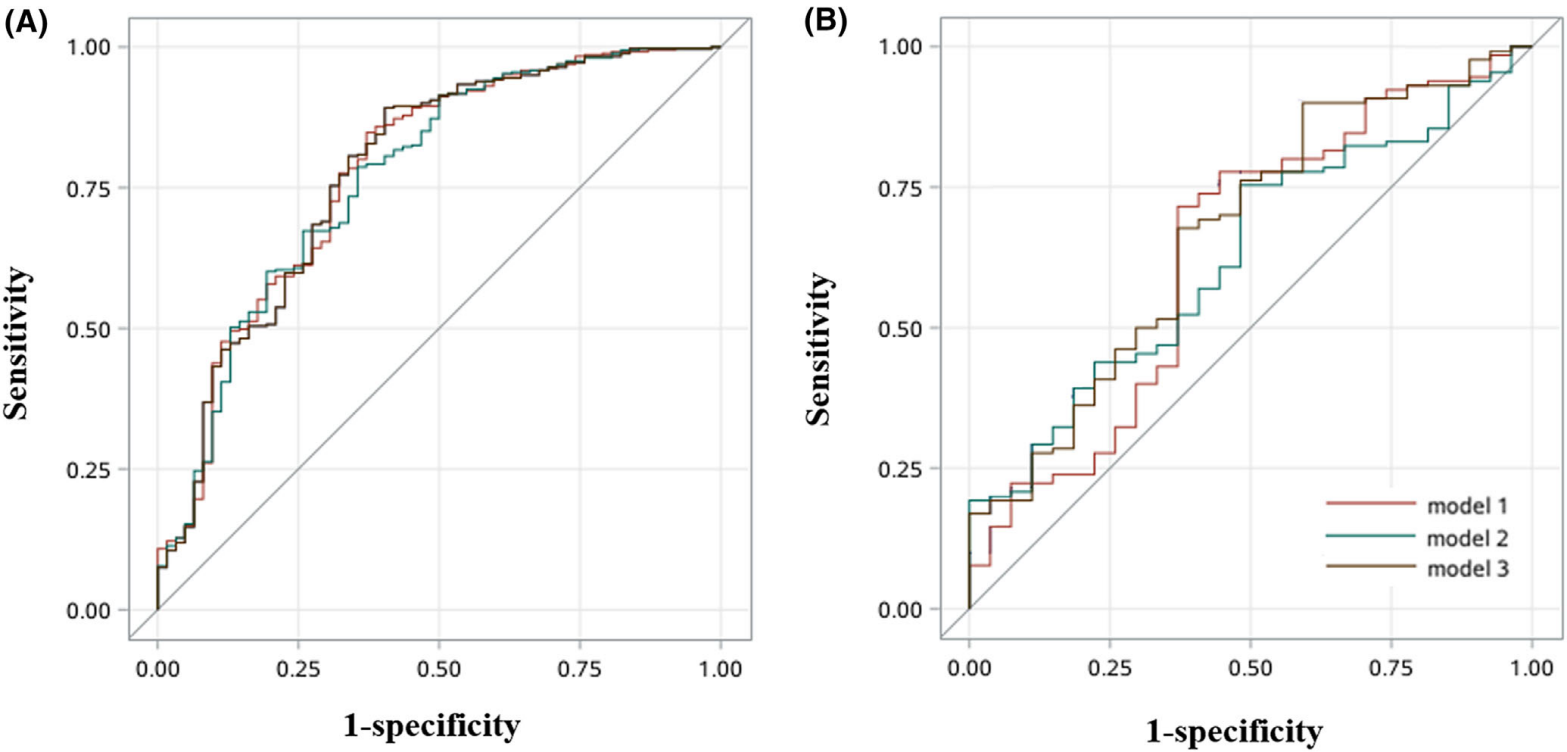
ROC curves for three OSAHS diagnostic models among high‐risk patients in training dataset (A) and validation dataset (B).

In both training dataset and validation dataset, the above three models presented similar discriminant ability; however, the best one is Model 1 with an AUC of 0.780 (0.711, 0.848), sensitivity of 0.848 (0.811, 0.885), specificity of 0.629 (0.509, 0.749), accuracy of 0.816 (0.779, 0.853), positive predictive value of 0.930 (0.903, 0.958), negative predictive value of 0.415 (0.315, 0.515), positive likelihood ratio of 2.286 (1.538, 3.034), and negative likelihood ratio of 0.242 (0.167, 0.316) in training dataset (Table 4). Even though, compared with other two models, the ROC curve and AUC were not statistically significant with *P*‐value = 0.5433 for Model 2 versus Model 1 and *P*‐value = 0.9518 for Model 3 versus Model 1 in training dataset (Figure 1). Similarly, in validation dataset, the comparisons were also non‐significant (all *P*‐value > 0.05).

**TABLE 4:** The comparison of discrimination of the diagnosis models in training and validation datasets.

Models	AUC (95% CI)	Sensitivity (95% CI)	Specificity (95% CI)	Accuracy (95% CI)	Positive predictive value (95% CI)	Negative predictive value (95% CI)	Positive LR (95% CI)	Negative LR (95% CI)
Training dataset								
Model 1	0.780 (0.711, 0.848)	0.848 (0.811, 0.885)	0.629 (0.509, 0.749)	0.816 (0.779, 0.853)	0.930 (0.903, 0.958)	0.415 (0.315, 0.515)	2.286 (1.538, 3.034)	0.242 (0.167, 0.316)
Model 2	0.770 (0.701, 0.839)	0.768 (0.726, 0.809)	0.646 (0.530, 0.762)	0.751 (0.711, 0.790)	0.930 (0.902, 0.957)	0.313 (0.235, 0.392)	2.170 (1.447, 2.892)	0.360 (0.268, 0.451)
Model 3	0.780 (0.711, 0.849)	0.889 (0.857, 0.922)	0.597 (0.475, 0.719)	0.846 (0.812, 0.881)	0.928 (0.901, 0.955)	0.481 (0.369, 0.592)	2.205 (1.533, 2.878)	0.186 (0.119, 0.252)
Validation dataset								
Model 1	0.637 (0.512, 0.762)	0.862 (0.802, 0.921)	0.296 (0.124, 0.469)	0.764 (0.698, 0.831)	0.855 (0.795, 0.915)	0.308 (0.130, 0.485)	1.224 (0.913, 1.536)	0.467 (0.130, 0.805)
Model 2	0.627 (0.518, 0.737)	0.746 (0.671, 0.821)	0.556 (0.368, 0.743)	0.713 (0.643, 0.784)	0.890 (0.831, 0.949)	0.313 (0.181, 0.444)	1.679 (0.951, 2.407)	0.457 (0.252, 0.662)
Model 3	0.668 (0.552, 0.783)	0.576 (0.407, 0.744)	0.065 (0.021, 0.108)	0.172 (0.113, 0.231)	0.141 (0.082, 0.199)	0.364 (0.163, 0.565)	0.616 (0.433, 0.798)	6.576 (1.452, 11.70)

*Note*: Model 1: adjusted for age, BMI, TB, syndrome of morning dry mouth or mouth breathing and high Berlin score. Model 2: adjusted for age, gender, BMI, TB, and syndrome of morning dry mouth or mouth breathing. Model 3: adjusted for age, gender, BMI, TB, syndrome of morning dry mouth or mouth breathing and high Berlin score. Training dataset: Model 2 versus Model 1: *P*‐value = 0.5433, Model 3 versus Model 1: *P*‐value = 0.9518. Validation dataset: Model 2 versus Model 1: *P*‐value = 0.7778, Model 3 versus Model 1: *P*‐value = 0.1171.

**FIGURE 2:**
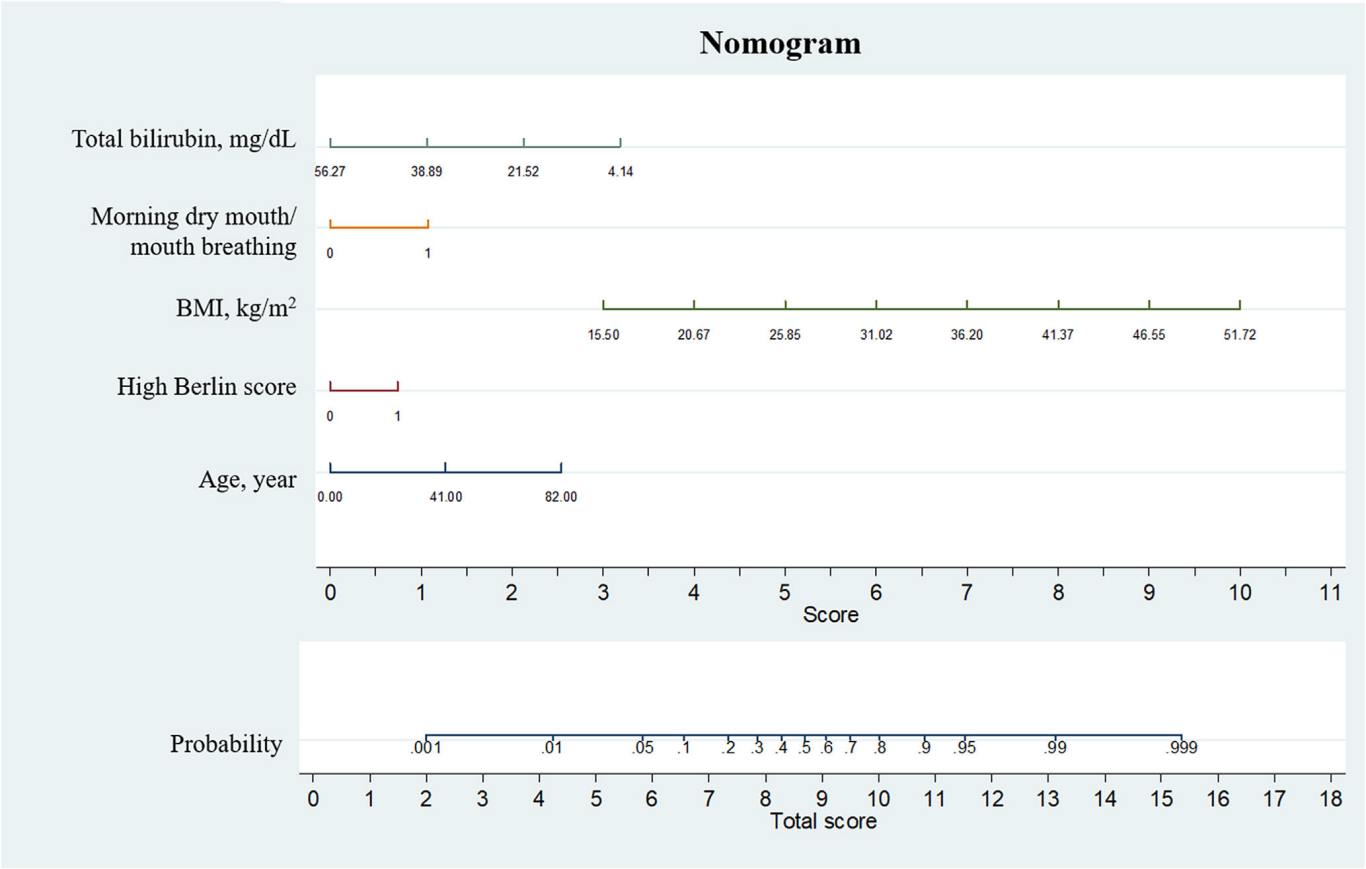
Nomogram model for OSAHS diagnosis among high‐risk OSAHS patients in training dataset.

We finally built the nomogram based on the Model 2 (Figure 2). The points assigned to each factor was on the score line and the total probability and score were on the bottom. For example, a patient aged 40 years with a BMI of 25 kg/m^2^, high Berlin score, with total bilirubin of 21.52 mg/dL, with a symptom of mouth breathing has an approximately 80% chance to be OSAHS.

## 4 DISCUSSION

Using the cross‐sectional data of 782 patients, we developed and validated a practical nomogram model to help us screen the OSAHS patient in a high‐risk Chinese population with a well discriminant ability. Our nomogram included five factors provides an efficient, convenient, and simple tool for clinical diagnosis of OSAHS.

Previous evidence suggested that the males have a high risk of OSA, the rate of males versus females in OSA patient populations was 8:1.^16^ However, in our study, after stepwise multivariable selection, the gender was not included into the final diagnostic model. The potential reason may be that the patients in our study were those high‐risk patients of OSAHS. Although the males were more likely to develop OSA than females, among those high‐risk patients, no matter males or females develop OSA in the similar risk. Additionally, in undiagnosed OSA populations, the ratio of males versus females was 2:1, which indicated that women with OSA are less likely to be evaluated and diagnosed.^6^ Moreover, evidence suggested that OSA in women may be diagnosed late in the course of the disease or may not be aggressively treated.^6^ Considering the gender difference in OSAHS, we additionally included gender into other models, but non‐significant differences from our final model (without gender) were detected in both training and validation datasets.

Age^17^, ^18^, ^19^ and BMI^16^, ^20^ have been confirmed as the critical risk factors of OSAHS, and all these two factors were included in our final model as a diagnostic factor. Our nomogram model indicated that BMI indicated a high contribution to OSAHS diagnosis. Previous evidence not only suggested that BMI increase results in OSAHS, nearly a 10% increase in weight was associated with a sixfold greater risk of developing OSA among persons initially free of OSA,^20^ but also the weight loss according to surgery or diet are significantly decrease in the severity of OSAHS.^16^ Besides that, the OSA patients with obesity would have a high risk of cardiovascular diseases in further.^21^ The underlying mechanism of obesity and OSAHS might be caused by the effects of intermittent hypoxia on adipose tissue.^22^, ^23^ Also, insufficient sleep may counteract with dysmetabolic changes.^24^ Moreover, both OSAHA and obesity induced the systemic pro‐inflammatory changes and finally enhanced the risk of cardiovascular diseases.^25^, ^26^ Older age adults (age ≥65) have a twofold to threefold higher prevalence of OSA than those aged less than 65 years.^17^, ^18^, ^19^ However, the under mechanism of the influence of age on OSAHS is still uncertain.

Bilirubin, an endogenous antioxidant in human vascular endothelial cells, provides an additional mechanism for hypoxic adaptation mediated through feedback inhibition of the heme biosynthetic pathway.^27^, ^28^ A meta‐analysis of 11 studies found an inverse relationship between bilirubin concentrations and atherosclerosis severity in men with a 6.5% decrease in cardiovascular events for every 1 mol/L increase in bilirubin.^29^ Among patients with OSAHS, the hyoxaemia would introduce a series of chain reactions in metabolism and changes in metabolism. Thus, the metabolism biomarkers might be the sensitivity response factors of OSAHS. This hypothesis has been supported by previous evidence,^30^ which found an increase in bilirubin levels of patients with OSA in the morning. Consistently, after the competition of multiple variables, total bilirubin levels were selected as a critical indicator for OSAHS in Chinese adults.

Morning dry mouth or mouth breathing also has been considered as an screening factors for sleep apnoea, because the dry mouth symptom significantly correlated with snoring and sleep apnoea.^31^ The specificity and diagnostic accuracy of the STOP‐Bang questionnaire have been improved by integrating dry mouth. Besides of other influence factors, such as hypertension and diabetes, due to the open‐mouth breathing during night sleep, the patients with OSA more frequently report a more typical dry mouth symptom in the morning. Our study reconfirmed that the morning dry mouth or mouth breathing might be a valuable and typical predictor for OSA.^32^

Using the data from a cross‐sectional study in Chinese adults, we develop a convenient, simple, and efficient diagnostic nomogram model, which could efficiently help clinicians to quickly discriminant the OSAH patients from those high‐risk population. All the risk factors were easily obtained, which might be have a wider applicability in practice. However, this study has several limitations. This is single centre study, which included the patients from one hospital and might include selection bias. Further need an external validation in other population, although we validated the model efficient and utility in a special training population form the same centre. We included many risk factors; however, there might be other diagnostic factors still not included, for example other saliva biomarkers^33^ and gene assessments.^34^, ^35^

Our study provided an efficient, convenient, and simple nomogram scale with five factors to help clinical screening OSAHS patients in those high‐risk population in China, which has a wider applicability in practice. However, due to the limits of cross‐sectional design and single centre study, the utility and feasibility need further reconfirmed in other population and verified by prospective studies.

## AUTHOR CONTRIBUTIONS

Jie Liu contributed to conceptualization. Feng Pang contributed to methodology. Xiaofeng Huang and Minmin Lin contributed to data collection and supervision. Xiangmin Zhang and Tianrun Liu contributed to project administration. Wenmin Deng contributed to data curation. Zhen Long contributed to reviewing and editing of the manuscript. All authors have read and approved the final manuscript.

## CONFLICT OF INTEREST STATEMENT

All authors declare that they have no conflict of interest.

## ETHICS STATEMENT

The study was approved by the Ethics Committee of The Sixth Affiliated Hospital Sun Yat‐sen University (Approval number: 2023ZSLYEC‐001). Consent to participate is waived due to it was an observational study.

## Data Availability

The datasets used and/or analysed during the current study are available from the corresponding author on reasonable request.

## References

[1] American Society of Anesthesiologists Task Force on Perioperative Management of patients with obstructive sleep apnea . Practice guidelines for the perioperative management of patients with obstructive sleep apnea: an updated report by the American Society of Anesthesiologists Task Force on perioperative management of patients with obstructive sleep apnea. Anesthesiology. 2014;120(2):268‐286. doi:10.1097/ALN.0000000000000053 24346178

[2] Senaratna CV , Perret JL , Lodge CJ , et al. Prevalence of obstructive sleep apnea in the general population: a systematic review. Sleep Med Rev. 2017;34:70‐81. doi:10.1016/j.smrv.2016.07.002 27568340

[3] Kuvat N , Tanriverdi H , Armutcu F . The relationship between obstructive sleep apnea syndrome and obesity: a new perspective on the pathogenesis in terms of organ crosstalk. Clin Respir J. 2020;14(7):595‐604. doi:10.1111/crj.13175 32112481

[4] Marin JM , Carrizo SJ , Vicente E , Agusti AG . Long‐term cardiovascular outcomes in men with obstructive sleep apnoea‐hypopnoea with or without treatment with continuous positive airway pressure: an observational study. Lancet. 2005;365(9464):1046‐1053. doi:10.1016/S0140-6736(05)71141-7 15781100

[5] Yaggi HK , Concato J , Kernan WN , Lichtman JH , Brass LM , Mohsenin V . Obstructive sleep apnea as a risk factor for stroke and death. N Engl J Med. 2005;353(19):2034‐2041. doi:10.1056/NEJMoa043104 16282178

[6] Young T , Finn L . Epidemiological insights into the public health burden of sleep disordered breathing: sex differences in survival among sleep clinic patients. Thorax. 1998;53(Suppl 3):S16‐S19. doi:10.1136/thx.53.2008.S16 10193355 PMC1765905

[7] Tasbakan MS , Gunduz C , Pirildar S , Basoglu OK . Quality of life in obstructive sleep apnea is related to female gender and comorbid insomnia. Sleep Breath. 2018;22(4):1013‐1020. doi:10.1007/s11325-018-1621-y 29352360

[8] Tarasiuk A , Greenberg‐Dotan S , Brin YS , Simon T , Tal A , Reuveni H . Determinants affecting health‐care utilization in obstructive sleep apnea syndrome patients. Chest. 2005;128(3):1310‐1314. doi:10.1378/chest.128.3.1310 16162723

[9] Tarasiuk A , Greenberg‐Dotan S , Simon‐Tuval T , Oksenberg A , Reuveni H . The effect of obstructive sleep apnea on morbidity and health care utilization of middle‐aged and older adults. J am Geriatr Soc. 2008;56(2):247‐254. doi:10.1111/j.1532-5415.2007.01544.x 18251815

[10] Ruaro B , Baratella E , Confalonieri M , Antonaglia C , Salton F . Editorial: obstructive sleep apnea syndrome (OSAS): what's new? Front Med. 2022;9:1009410. doi:10.3389/fmed.2022.1009410 PMC9521371 36186790

[11] Kapur V , Strohl KP , Redline S , Iber C , O'Connor G , Nieto J . Underdiagnosis of sleep apnea syndrome in U.S. communities. Sleep Breath. 2002;6(2):49‐54. doi:10.1055/s-2002-32318 12075479

[12] Kapur VK , Auckley DH , Chowdhuri S , et al. Clinical practice guideline for diagnostic testing for adult obstructive sleep apnea: an American Academy of Sleep Medicine Clinical Practice Guideline. J Clin Sleep Med. 2017;13(03):479‐504. doi:10.5664/jcsm.6506 28162150 PMC5337595

[13] Antonaglia C , Vidoni G , Contardo L , et al. Low arousal threshold estimation predicts failure of mandibular advancement devices in obstructive sleep apnea syndrome. Diagnostics. 2022;12(10):2548. doi:10.3390/diagnostics12102548 36292237 PMC9600433

[14] Tawaranurak K , Kamolphiwong S , Sae‐Wong S , et al. Validity of a new prediction model to identify patients at risk for obstructive sleep apnea hypopnea syndrome. Ear Nose Throat J. 2023;102(1):52‐57. doi:10.1177/0145561320986045 33393817

[15] Luo J , Huang R , Zhong X , Xiao Y , Zhou J . STOP‐bang questionnaire is superior to Epworth sleepiness scales, Berlin questionnaire, and STOP questionnaire in screening obstructive sleep apnea hypopnea syndrome patients. Chin Med J (Engl). 2014;127(17):3065‐3070. 25189946

[16] Young T , Peppard PE , Gottlieb DJ . Epidemiology of obstructive sleep apnea: a population health perspective. Am J Respir Crit Care Med. 2002;165(9):1217‐1239. doi:10.1164/rccm.2109080 11991871

[17] Bixler EO , Vgontzas AN , Ten Have T , Tyson K , Kales A . Effects of age on sleep apnea in men: I. Prevalence and severity. Am J Respir Crit Care Med. 1998;157(1):144‐148. doi:10.1164/ajrccm.157.1.9706079 9445292

[18] Bixler EO , Vgontzas AN , Lin HM , et al. Prevalence of sleep‐disordered breathing in women: effects of gender. Am J Respir Crit Care Med. 2001;163(3):608‐613. doi:10.1164/ajrccm.163.3.9911064 11254512

[19] Young T , Shahar E , Nieto FJ , et al. Predictors of sleep‐disordered breathing in community‐dwelling adults: the Sleep Heart Health Study. Arch Intern Med. 2002;162(8):893‐900. doi:10.1001/archinte.162.8.893 11966340

[20] Peppard PE , Young T , Palta M , Dempsey J , Skatrud J . Longitudinal study of moderate weight change and sleep‐disordered breathing. Jama. 2000;284(23):3015‐3021. doi:10.1001/jama.284.23.3015 11122588

[21] Bock JM , Vungarala S , Karim S , Somers VK . Obstructive sleep apnea as a cardiovascular risk factor‐beyond CPAP. Can J Cardiol. 2021;37(5):756‐765. doi:10.1016/j.cjca.2021.01.027 33610689 PMC8102326

[22] Gileles‐Hillel A , Almendros I , Khalyfa A , et al. Prolonged exposures to intermittent hypoxia promote visceral white adipose tissue inflammation in a murine model of severe sleep apnea: effect of normoxic recovery. Sleep. 2017;40(3):zsw074. doi:10.1093/sleep/zsw074 28329220

[23] Ryan S , Arnaud C , Fitzpatrick SF , Gaucher J , Tamisier R , Pépin JL . Adipose tissue as a key player in obstructive sleep apnoea. Eur Respir Rev. 2019;28(152):190006. doi:10.1183/16000617.0006-2019 31243096 PMC9488701

[24] Henst RHP , Pienaar PR , Roden LC , Rae DE . The effects of sleep extension on cardiometabolic risk factors: a systematic review. J Sleep Res. 2019;28(6):e12865. doi:10.1111/jsr.12865 31166059

[25] Poroyko VA , Carreras A , Khalyfa A , et al. Chronic sleep disruption alters gut microbiota, induces systemic and adipose tissue inflammation and insulin resistance in mice. Sci Rep. 2016;6(1):35405. doi:10.1038/srep35405 27739530 PMC5064361

[26] Chaurasia B , Kaddai VA , Lancaster GI , et al. Adipocyte ceramides regulate subcutaneous adipose Browning, inflammation, and metabolism. Cell Metab. 2016;24(6):820‐834. doi:10.1016/j.cmet.2016.10.002 27818258

[27] Jones DP . Oxygen conformance and cellular regulation. LUNG BIOLOGY IN HEALTH AND DISEASE. Vol. 105; 1997:49‐66.

[28] Ferreira GC , Dailey HA . Mouse protoporphyrinogen oxidase. Kinetic parameters and demonstration of inhibition by bilirubin. Biochem J. 1988;250(2):597‐603. doi:10.1042/bj2500597 2451512 PMC1148896

[29] Novotný L , Vítek L . Inverse relationship between serum bilirubin and atherosclerosis in men: a meta‐analysis of published studies. Exp Biol Med (Maywood). 2003;228(5):568‐571. doi:10.1177/15353702-0322805-29 12709588

[30] Chin K , Ohi M , Shimizu K , et al. Increase in bilirubin levels of patients with obstructive sleep apnea in the morning—a possible explanation of induced Heme Oxygenase‐1. Sleep. 2001;24(2):218‐223. doi:10.1093/sleep/24.2.218 11247059

[31] Zhang C , Shen Y , Liping F , Ma J , Wang GF . The role of dry mouth in screening sleep apnea. Postgrad Med J. 2021;97(1147):294‐298. doi:10.1136/postgradmedj-2020-137619 32913036

[32] Oksenberg A , Froom P , Melamed S . Dry mouth upon awakening in obstructive sleep apnea. J Sleep Res. 2006;15(3):317‐320. doi:10.1111/j.1365-2869.2006.00527.x 16911034

[33] Traxdorf M , Wendler O , Tziridis K , Bauer J , Scherl C . S100B in serum and saliva: a valid invasive or non‐invasive biomarker in obstructive sleep apnea? Eur Rev Med Pharmacol Sci. 2016;20(22):4766‐4774. 27906424

[34] Chen H , Hu K , Zhu J , et al. Polymorphisms of the 5‐hydroxytryptamine 2A/2C receptor genes and 5‐hydroxytryptamine transporter gene in Chinese patients with OSAHS. Sleep Breath. 2013;17(4):1241‐1248. doi:10.1007/s11325-013-0829-0 23494654

[35] Xu H , Guan J , Yi H , Yin S . A systematic review and meta‐analysis of the association between serotonergic gene polymorphisms and obstructive sleep apnea syndrome. PLoS ONE. 2014;9(1):e86460. doi:10.1371/journal.pone.0086460 24475124 PMC3903532

